# Insulin inhibits glucagon release by SGLT2-induced stimulation of somatostatin secretion

**DOI:** 10.1038/s41467-018-08193-8

**Published:** 2019-01-11

**Authors:** Elisa Vergari, Jakob G. Knudsen, Reshma Ramracheya, Albert Salehi, Quan Zhang, Julie Adam, Ingrid Wernstedt Asterholm, Anna Benrick, Linford J. B. Briant, Margarita V. Chibalina, Fiona M. Gribble, Alexander Hamilton, Benoit Hastoy, Frank Reimann, Nils J. G. Rorsman, Ioannis I. Spiliotis, Andrei Tarasov, Yanling Wu, Frances M. Ashcroft, Patrik Rorsman

**Affiliations:** 1 Radcliffe Department of Medicine, Oxford Centre for Diabetes, Endocrinology and Metabolism, Churchill Hospital, Oxford, OX3 7LE UK; 2 Department of Physiology, Institute of Neuroscience and Physiology, University of Göteborg, Box 430, Göteborg, SE40530 Sweden; 3 Cambridge Institute of Metabolic Science and MRC Metabolic Diseases Unit, University of Cambridge School of Clinical Medicine, Cambridge Biomedical Campus, Hills Road, Cambridge, CB2 0QQ UK; 4 Oxford National Institute for Health Research, Biomedical Research Centre, Churchill Hospital, Oxford, OX3 7LE UK; 5 Department of Physiology, Anatomy and Genetics, Henry Wellcome Centre for Gene Function, University of Oxford, Parks Road, Oxford, OX1 3PT UK

## Abstract

Hypoglycaemia (low plasma glucose) is a serious and potentially fatal complication of insulin-treated diabetes. In healthy individuals, hypoglycaemia triggers glucagon secretion, which restores normal plasma glucose levels by stimulation of hepatic glucose production. This counterregulatory mechanism is impaired in diabetes. Here we show in mice that therapeutic concentrations of insulin inhibit glucagon secretion by an indirect (paracrine) mechanism mediated by stimulation of intra-islet somatostatin release. Insulin’s capacity to inhibit glucagon secretion is lost following genetic ablation of insulin receptors in the somatostatin-secreting δ-cells, when insulin-induced somatostatin secretion is suppressed by dapagliflozin (an inhibitor of sodium-glucose co-tranporter-2; SGLT2) or when the action of secreted somatostatin is prevented by somatostatin receptor (SSTR) antagonists. Administration of these compounds in vivo antagonises insulin’s hypoglycaemic effect. We extend these data to isolated human islets. We propose that SSTR or SGLT2 antagonists should be considered as adjuncts to insulin in diabetes therapy.

## Abstract (web summary)

Impaired glucagon secretion in patients with diabetes causes hypoglycemia. Here the authors show that therapeutic concentrations of insulin inhibit alpha-cell glucagon secretion by stimulating delta-cell insulin receptor and the release of somatostatin. Blocking somatostatin secretion or action ameliorates this effect.

## Introduction

Plasma glucose is maintained by a tug-of-war between the hypoglycaemic effect of insulin and the hyperglycaemic effect of glucagon. Under normal conditions, the plasma glucose is maintained at ≈5 mM in man. The benefits of good glycaemic control in diabetic patients are well known: it prevents or delays diabetic retinopathy, nephropathy and neuropathy^1^.

Two major forms of diabetes are recognised: type 1 (T1D) has a young age of onset and results in loss of insulin-secreting β cells and a lifetime requirement for insulin replacement therapy. Type 2 diabetes (T2D) largely affects older subjects and involves impaired insulin secretion and/or action. In both forms of diabetes, the hyperglycaemic effects of insulin deficiency are aggravated by hypersecretion of glucagon^2^. Therapy includes drugs to stimulate insulin release but when this fails, insulin injections are required. However, accurate administration of insulin to maintain normoglycaemia is difficult; too little will not regulate glucose and too much exogenous insulin may produce hypoglycaemia.

Hypoglycaemia results in glucose deficiency in the brain, coma and (if not alleviated) ultimately death. In normal situations, hypoglycaemia would trigger a counter-regulatory response in the α cells (stimulation of glucagon release and increased hepatic glucose production) but this does not occur in many T1D and some T2D patients^3^. Patients with T1D experience on average two episodes of symptomatic hypoglycaemia every week^4^ and it has been estimated that up to 10% of these patients die of iatrogenic hypoglycaemia^5^. Thus, hypoglycaemia is ‘the limiting factor in diabetes therapy’^6^ and, if it were not for hypoglycaemia, diabetes could be easily managed simply by increasing the insulin dose until normoglycaemia is restored.

Pancreatic islets are complex structures consisting of several types of endocrine cell. In addition to the insulin-producing β cells and glucagon-secreting α cells, islets also contain a small number (5–10%) of somatostatin-secreting δ cells^7^. The regulation of somatostatin release is complex and involves a crosstalk between paracrine and intrinsic effects^8^. The δ cells are electrically excitable and somatostatin secretion is associated with increased action potential firing involving activation of voltage-gated Ca^2+^ channels. The increase in cytoplasmic Ca^2+^ resulting from plasmalemmal Ca^2+^ entry is amplified by Ca^2+^-induced Ca^2+^ release (CICR) from intracellular Ca^2+^ stores^9^. Somatostatin is a paracrine inhibitor of both insulin and glucagon^10–14^. Accumulating evidence suggests that increased somatostatin signalling, via suppression of glucagon secretion, results in the loss of appropriate counter regulation during insulin-induced hypoglycaemia^15,16^. However, the link (if any) between insulin therapy and the loss of counter regulation remains obscure.

Here we have investigated the regulation of glucagon secretion by insulin in mouse and human islets. We show that insulin inhibits glucagon secretion by a paracrine effect mediated by stimulation of somatostatin secretion rather than a direct effect on the α cells. These findings highlight the importance of the intra-islet paracrine crosstalk and suggest that therapeutically targeting somatostatin secretion or action may restore counter-regulatory glucagon secretion and thus minimise the risk of fatal hypoglycaemia.

## Results

### Insulin stimulates somatostatin secretion

**Fig. 1:**
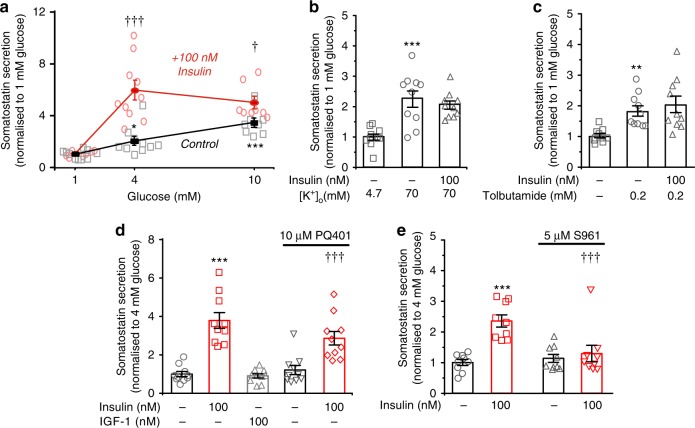
Insulin stimulates somatostatin secretion. **a** Somatostatin release at 1, 4 and 10 mM glucose in the absence or presence of 100 nM insulin (*n* = 8–10 experiments using six male mice). ^†^*p* < 0.05, ^†††^*p* < 0.001 vs the same glucose concentration but in the absence of insulin, one-way ANOVA followed by Dunnet’s post hoc test. Glucose (4 and 20 mM) also stimulates somatostatin secretion compared with 1 mM in both the absence and presence of insulin (*p* < 0.05 or better, not indicated for clarity). **b**, **c** Lack of effect of insulin on somatostatin secretion elicited by high [K^+^]_o_ (70 mM at 4 mM glucose; **b**) and by the K_ATP_ channel blocker tolbutamide (0.2 mM at 1 mM glucose; **c**). When [K^+^]_o_ was increased, Na^+^ was correspondingly reduced to maintain iso-osmolarity. **p < 0.01 vs 1 mM glucose alone (*n* = 10 experiments/5 male mice), one-way ANOVA followed by Dunnet’s post hoc test. **d** Effects of insulin or IGF-1 on somatostatin secretion at 4 mM glucose in the absence and presence of the IGF-1R inhibitor PQ401 (*n* = 10 experiments using six male mice). ***p < 0.001 vs control (no insulin); ^†††^*p* < 0.001 vs control in the presence of PQ401, one-way ANOVA followed by Dunnet’s post hoc test. **e** Effects of insulin and the insulin receptor antagonist S961 on somatostatin release in isolated mouse islets incubated at 4 mM glucose (*n* = 9 experiments/3 male mice). ***p < 0.001 vs no insulin. ^†††^*p* < 0.001 vs 100 nM insulin in the absence of S961, one-way ANOVA followed by Dunnet’s post hoc test. Responses have been normalised to somatostatin secretion at 1 mM or 4 mM glucose as indicated. Data are presented as dot plots of individual experiments and/or mean values ± S.E.M. of all experiments with the experimental series

In preliminary experiments, we found that insulin stimulates somatostatin secretion in isolated pancreatic islets. We examined the glucose dependence of insulin’s stimulatory effect on somatostatin release. It was negligible at 1 mM glucose and limited to 50% at 10 mM glucose. However, at 4 mM glucose, insulin enhanced somatostatin release by >200% (Fig. 1a). Insulin had no stimulatory effect when applied in the presence of 70 mM K^+^ (Fig. 1b), a condition that depolarises the δ cells to −11 ± 1 mV (mean value ± standard error of the mean of six experiments: not shown), or when tested in the presence of 0.2 mM of the K_ATP_ channel blocker tolbutamide (Fig. 1c), which initiates continuous action potential firing in δ cells^17^. The effects of insulin on somatostatin release were not mimicked by insulin-like growth factor 1 (IGF-1), resistant to the IGF-1 receptor antagonist PQ401^18^ (Fig. 1d) but abolished in the presence of the insulin receptor antagonist S961 (Fig. 1e). Collectively, these observations suggest that insulin exerts its effect on somatostatin secretion by binding to insulin receptors, is without stimulatory effect at low glucose, exerts its greatest stimulatory effect at glucose concentrations slightly above the threshold for initiation of somatostatin secretion, is ineffective when the K_ATP_ channels are fully closed and requires a negative membrane potential.

### Insulin’s glucagonostatic mediated by somatostatin

**Fig. 2:**
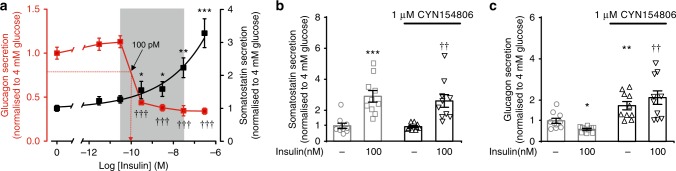
Insulin exerts its glucagonostatic effect by somatostatin. **a** Effects of increasing concentrations of insulin (3 pM, 30 pM, 300 pM, 3 nM, 30 nM, 300 nM; logarithmic abscissa) on glucagon (red) and somatostatin (black) secretion in the presence of 4 mM glucose (*n* = 10 experiments/6 male mice). Arrow indicates IC_50_ for inhibitory effect of insulin on glucagon secretion (~100 pM). *p < 0.05, **p < 0.01 and ***p < 0.001 and ^†††^*p* < 0.001 for the effects of insulin on somatostatin and glucagon secretion vs 4 mM glucose alone, respectively, one-way ANOVA followed by Dunnet’s post hoc test. **b** Somatostatin secretion at 4 mM glucose in the absence and presence of insulin and/or CYN154806 (*n* = 10 experiments//6 male mice). ***p < 0.001 vs 4 mM glucose; ^††^*p* < 0.005 vs 4 mM glucose and CYN154806, one-way ANOVA followed by Dunnet’s post hoc test. **c** As in **b** but glucagon is measured (*n* = 10 experiments/6 male mice). Secretion has been normalised to secretion rates at 4 mM glucose. *p < 0.05 and **p < 0.01 vs 4 mM glucose; ^††^*p* < 0.005 vs 4 mM glucose and insulin. Data are presented as dot plots of individual experiments and/or mean values ± S.E.M. of all experiments with the experimental series

Somatostatin is a powerful inhibitor of glucagon secretion^10^. We hypothesised that insulin’s glucagonostatic action could be mediated by somatostatin. We first measured the effects of increasing concentrations of insulin on glucagon and somatostatin release in isolated mouse pancreatic islets exposed to 4 mM glucose (Fig. 2a). Insulin inhibited glucagon secretion in a dose-dependent manner (IC_50_ ≈100 pM) with nearly maximal inhibition at 300 pM. This was echoed by a concentration-dependent stimulation of somatostatin secretion that was first detectable at 300 pM of insulin and that did not saturate even at concentrations as high as 0.3 μM. For comparison, plasma insulin levels in non-diabetic individuals range between ~50 pM and 450 pM and may be as high as 8 nM in insulin-treated type 1 diabetic patients^19^.

We next examined whether the inhibitory effect of insulin on glucagon secretion are mediated by somatostatin using the somatostatin receptor (SSTR) antagonist CYN154806. In the presence of 1 μM of CYN154806, the inhibitory effect of insulin on glucagon secretion was abolished, whereas the stimulatory effect on somatostatin secretion was unaffected (Fig. 2b, c). At the concentration used, CYN154806 is expected to block both SSTR2 and SSTR3^20^, the functionally important SSTRs in human^21^ and mouse α cells^22^. CYN154806 also increased glucagon secretion measured at 4 mM glucose by ~60%, an effect we attribute to the relief from paracrine suppression by somatostatin released at this glucose concentration.

### No glucagonostatic effect of insulin in SIRKO mice

**Fig. 3:**
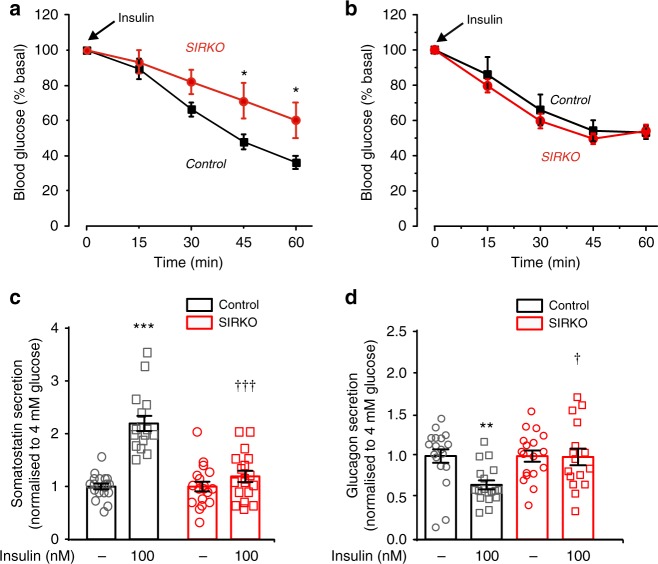
Insulin’s glucagonostatic effect mediated by InsR in δ cells. **a**, **b** Insulin tolerance test. Plasma glucose concentrations following injection of insulin (at *t* = 0; 1.5 U/kg) in female (**a**) and male (**b**) controls (black; *n* = 10 female and 6 male mice) and δ-cell-specific insulin receptor knockout (SIRKO, red; *n* = 9 female and 6 male mice) mice. *p < 0.05 vs control, two-way ANOVA followed by Sidak’s post hoc test. **c**, **d** Somatostatin (**c**) and glucagon secretion (**d**) at 4 mM glucose in the absence or presence of insulin (100 nM) in islets from control (black; *n* = 20/4 mice) or SIRKO (red: *n* = 18 with insulin and 20 without insulin/4 mice) mice. Responses have been normalised to secretion at 4 mM glucose. **p < 0.01, ***p < 0.001 vs 4 mM glucose; ^†^*p* < 0.05 and ^†††^*p* < 0.001 vs 4 mM glucose and 100 nM insulin in control islets, two-way ANOVA followed by Sidak’s post hoc test. Data are presented as dot plots of individual experiments and/or mean values ± S.E.M. of all experiments with the experimental series

We tested the systemic role of insulin-induced somatostatin release by generating somatostatin-secreting δ-cell insulin receptor knockout (SIRKO) mice (Supplementary Fig. 2a, b). SIRKO mice were normal weight and normoglycaemic (Supplementary Fig. 2c, d). However, female SIRKO mice were much less insulin sensitive than female control mice (Fig. 3a). No difference in insulin sensitivity was detected between male control and SIRKO mice (Fig. 3b).

Increasing glucose from 1 to 4 mM stimulated somatostatin secretion and inhibited glucagon secretion in control and SIRKO islets (Supplementary Fig. 3a, b). In control islets, insulin (100 nM) stimulated somatostatin secretion by > 100% when tested at 4 mM glucose but was without effect in SIRKO islets (Fig. 3c). The loss of insulin-induced somatostatin release in SIRKO islets correlated with the complete abrogation of insulin’s glucagonostatic effect (Fig. 3d). We point out that the α cells in SIRKO mice retain their insulin receptors. The failure of insulin to affect glucagon secretion in SIRKO mice therefore suggests that activation of insulin receptors in α cells does not directly inhibit glucagon secretion. There were no differences between the effects of glucose or insulin on glucagon and somatostatin secretion in isolated islets from male and female SIRKO mice (Supplementary Fig. 3c-f). For this reason, hormone secretion data from male and female mice were pooled. Thus, the differences observed between male and female SIRKO mice in vivo are therefore likely to reflect systemic rather than intra-islet mechanisms. Interestingly, the counter-regulatory response also shows a sex difference in humans^23,24^.

### Insulin increases [Ca^2+^]_i_ in δ cells

**Fig. 4:**
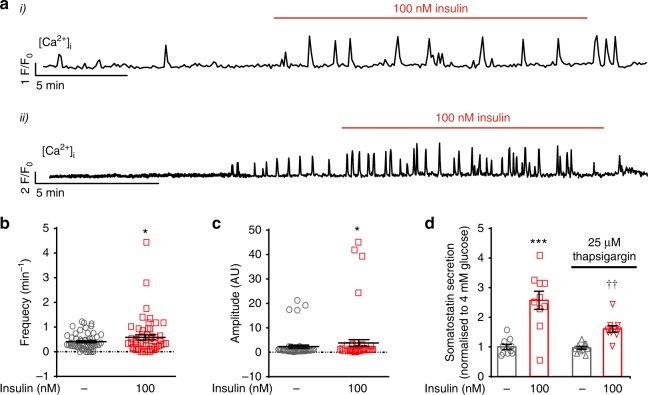
Effect of insulin on cytosolic Ca^2+^. **a** Effects of insulin on [Ca^2+^]_i_ in δ cells in intact islets exposed to 4 mM glucose. Two representative traces are shown. **b**, **c** Frequency (**b**) and amplitude (**c**) of [Ca^2+^]_i_ oscillations at 4 mM glucose in the absence and presence of insulin. *p < 0.05 vs 4 mM glucose (*n* = 54 δ cells in 11 islets from 8 mice of both sexes), Student’s *t*-test. **d** Effects of insulin (100 nM) on somatostatin secretion at 4 mM glucose in the absence and presence of the SERCA inhibitor thapsigargin (25 μM). ***p < 0.001 vs 4 mM glucose; ^††^*p* < 0.01 vs 4 mM glucose and insulin in the absence of thapsigargin (*n* = 10 experiments/4 male mice), one-way ANOVA followed by Sidak’s post hoc test. Data in **b**–**d** are presented as dot plots of individual experiments and/or mean values ± S.E.M. of all experiments with the experimental series

Glucose-induced somatostatin release is a Ca^2+^-dependent process that depends on CICR^9^. We investigated the effect of insulin on δ-cell [Ca^2+^]_i_ in islets expressing the genetically encoded Ca^2+^ indicator GCaMP3 under the control of the somatostatin promoter. In islets exposed to 4 mM glucose, ~50% of δ cells exhibited spontaneous [Ca^2+^]_i_ oscillations and application of insulin (100 nM) increased their frequency. Examples from two representative cells are shown in Fig. 4a. Insulin did not evoke [Ca^2+^]_i_ oscillations in δ cells that were not spontaneously active. Insulin increased the frequency and amplitude of these oscillations by 40% and 80%, respectively (Fig. 4b, c).

Despite increasing [Ca^2+^]_i_, insulin had no consistent effect on δ-cell action potential firing when tested at 4 mM glucose (Supplementary Fig. 4a-c). Close inspection of the measurements revealed that a small depolarisation (1–3 mV) was observed in four of the seven experiments (~55%) but even in these cells there was no consistent increase in action potential frequency. This suggests that the stimulatory effects of insulin on [Ca^2+^]_i_ principally involves mobilisation of Ca^2+^ from intracellular stores. Indeed, insulin-stimulated somatostatin secretion was reduced in the presence of thapsigargin, an inhibitor of the Ca^2+^-ATPase of the sarco/endoplasmic reticulum (SERCA) (Fig. 4d).

### SGLT2-dependent stimulation of somatostatin secretion

It has been reported that sodium-glucose co-tranporter-2 (SGLT2) inhibitors stimulate glucagon secretion both in vitro and in vivo^25–29^. We hypothesised that the stimulatory effect of insulin on somatostatin secretion is mediated by SGLT2, which is activated by insulin^30^, and that this suppresses glucagon secretion by a paracrine effect. At the mRNA level, *Sglt2*/*SGLT2* is expressed at low levels in mouse δ cells^22^. Using immunocytochemistry, we found that 58% and 33% of the somatostatin-positive cells were also positive for SGLT2 in mouse and human islet cell preparations, respectively. The corresponding values in somatostatin-negative cells (mainly α or β cells) were 8% and 13% (Supplementary Fig. 5).

**Fig. 5:**
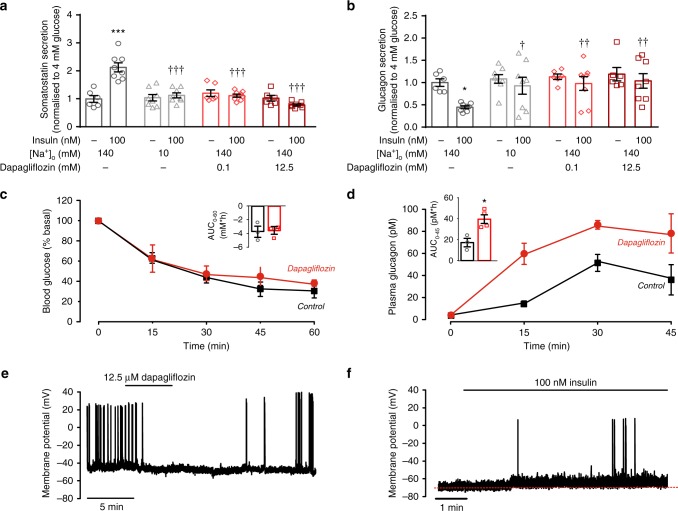
Insulin activates SGLT2 in δ cells. **a**, **b** Somatostatin (**a**) and glucagon secretion (**b**) at 4 mM glucose in the absence or presence of insulin at normal or lowered (10 mM) extracellular Na^+^ ([Na^+^]_o_) with or without dapagliflozin as indicated. Responses have been normalised to secretion at 4 mM glucose. * *p* < 0.05, ***p < 0.001 vs 4 mM glucose alone; ^†^*p* < 0.05, ^††^*p* < 0.05 ^†††^*p* < 0.001 for comparisons with 4 mM glucose under control conditions (black bar). Data are based on 6–8 experiments using islets from three male mice). Statistical analyses were performed by one-way ANOVA followed by Dunnett’s post hoc test. **c**, **d** Insulin tolerance test. Plasma glucose (**c**) and glucagon (**d**) concentrations following intraperitoneal (*ip*) injection of insulin (at *t* = 0; 0.5 U/kg) in male mice (age: 9 wks) followed by injection of dapagliflozin (10 mg/kg, ip: red) or vehicle (5% DMSO in sterile PBS, *ip*; black). (see Supplementary Fig. 5 for corresponding data in female mice). In **c**, **d**, insets summarise effects of dapagliflozin on plasma glucose and glucagon expressed as the area under the curve (AUC) during the first 45 min. *p < 0.05 vs control (no dapagliflozin) by Student’s *t*-test (4 mice for each condition). **e** Effects of the SGLT2 inhibitor dapagliflozin (12.5 μM) on δ-cell action potential firing in the presence of 4 mM glucose and 100 nM insulin. Dapagliflozin was applied as indicated above the trace (representative of *n* = 4 δ cells in four islets from three mice of both sexes). **f** Effects of insulin in the presence of 19 mM of the non-metabolisable glucose analogue α-methyl-d-glucopyranoside (αMDG) on δ-cell action potential firing (*n* = 5 cells from three mice of both sexes). Red dotted line indicates membrane potential before addition of insulin. Data in **a**–**d** are presented as dot plots of individual experiments and/or mean values ± S.E.M. of all experiments with the experimental series

We tested the functional role of SGLT2 in insulin-induced somatostatin secretion (Fig. 5a). The stimulatory effect of insulin was abolished when extracellular Na^+^ was reduced from the normal 140 mM to 10 mM. Similar inhibitory effects were produced by application of the SGLT2 inhibitor dapagliflozin at 0.1 and 12.5 μM (the lower concentration should be specific for SGLT2, whereas the higher concentration will also inhibit SGLT1). Notably, none of these procedures affected somatostatin secretion at 4 mM glucose alone.

The inhibitory effects of Na^+^ omission and pharmacological inhibition of SGLT2 on insulin-induced somatostatin secretion correlated with the loss of insulin’s glucagonostatic effect. Importantly, none of these experimental conditions affected glucagon secretion in the absence of insulin (i.e., at 4 mM glucose alone; Fig. 5b).

We performed insulin tolerance tests in the absence and presence of dapagliflozin. Regardless of whether co-administered with dapagliflozin or not, insulin reduced plasma glucose by ~60% (Fig. 5c). Despite the weak effect on plasma glucose levels, dapagliflozin strongly stimulated glucagon secretion (Fig. 5d). These experiments were performed in male mice but similar effects were observed in female mice (Supplementary Fig. 6a-b). We point out that only 5% of somatostatin in plasma is derived from the pancreatic δ cells^8^ and most assays (including the one we use) cannot distinguish between pancreatic islet- and gut-derived somatostatin. It was therefore not attempted to correlate the increased plasma glucagon to plasma somatostatin.

To exclude the possibility that dapagliflozin acts by a direct effect on the α cell, we tested the effects of this SGLT2 inhibitor in SIRKO islets. Dapagliflozin only affected somatostatin and glucagon secretion in control islets whereas it had no effect in SIRKO islets (Supplementary Fig. 7a-b).

### SGLT2-dependent δ-cell electrical activity

SGLT2 is electrogenic (it co-transports Na^+^ and glucose with a stoichiometry of 1:1)^31^ and insulin-induced stimulation of its activity should accordingly be expected to depolarise the cell. Indeed, when dapagliflozin was applied in the simultaneous presence of 100 nM insulin and 4 mM glucose, it repolarised three out of four δ cells by an average of 3 ± 1 mV (*n* = 4; *p* < 0.05 by paired Student’s *t*-test) and reduced action potential firing from 0.5 ± 0.2 to 0.2 ± 0.1 Hz (*p* < 0.01 by paired Student’s *t*-test) (Fig. 5e). Application of 19 mM of α-methyl-d-glucopyranoside (αMDG), a non-metabolisable glucose analogue transported by SGLTs at 1 mM glucose depolarised the δ cell by an average of 2 ± 1 mV (*n* = 5) but did not initiate action potential firing. Addition of insulin the continued presence of αMDG resulted in a further depolarisation of 4 ± 2 mV (*n* = 5; *p* = 0.05 by paired Student’s *t*-test) and evoked action potential firing in two of the cells (Fig. 5f). The input resistance of δ cells exposed to 1 mM glucose averaged 3.2 ± 0.8 GΩ (*n* = 10). From Ohm’s law, we estimate that the operation of SGLT2 in δ cells gives rise to a current as small as ~1 pA. The small amplitude of the SGLT2-dependent current is consistent with the reported low mRNA expression of *Slc5a2*^22^.

We monitored the increase in cytoplasmic Na^+^ ([Na^+^]_i_) that should be associated with SGLT2-mediated glucose entry. When insulin was applied in the presence of 19 mM αMDG, [Na^+^]_i_ increased in 40% of the δ cells (Supplementary Fig. 8a-b). These may correspond to the δ cells that express SGLT2.

### Insulin’s glucagonostatic effect enhanced in diabetes

**Fig. 6:**
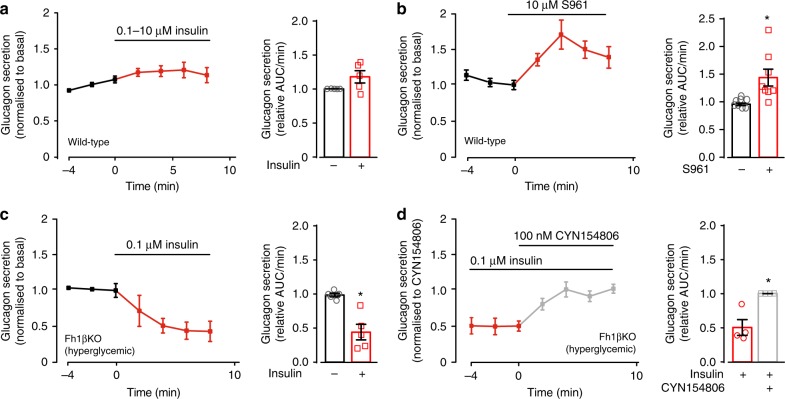
Effects of insulin in the perfused mouse pancreas. **a** Glucagon secretion measured before and after addition of insulin using the perfused mouse pancreas preparation. For these experiments, wild-type mice were used. In each experiment, glucagon secretion was normalised to that at 4 mM glucose prior to the addition of insulin (100 nM in three experiments and 10 μM in two experiments: responses have been pooled for display; female mice were used). **b** As in **a** but the insulin receptor antagonist S961 was used instead of insulin (*n* = 7 female mice). **c** As in **a** but experiments were performed in hyperglycaemic/diabetic Fh1βKO mice and using insulin at a concentration of 100 nM (*n* = 8 mice of both sexes). In **a**-**c**, the black bars represent mean glucagon secretion measured during the 6 min preceding the addition of insulin or S961. **d** As in (**c**) but CYN154806 was included as indicated (*n* = 4 diabetic Fh1βKO mice of both sexes). Glucagon secretion has been normalised to that in the presence of CYN154806 (measured at steady-state, 4–8 min after the addition; *t* = 4–8 min). The red and bars represent glucagon secretion in the presence of insulin alone and following the addition of CYN154806, respectively. Statistical significances were analysed by Student’s *t*-test. *p < 0.05. Data are presented as dot plots of individual experiments and/or mean values ± S.E.M. of all experiments with the experimental series

We used the perfused mouse pancreas preparation to explore the effects of insulin on δ cells in the intact pancreas. Under these experimental conditions, the pancreas is perfused in situ at the physiological rate (0.25 ml/min) and direction. Surprisingly, insulin (100 nM–10 μM) consistently failed to affect glucagon secretion when tested in the perfused mouse pancreas (Fig. 6a).

The lack of effect of exogenous insulin on glucagon secretion in the perfused pancreases contrasts to the strong inhibition seen in superficial islets and may indicate that glucagon secretion is already maximally suppressed by endogenous insulin released from neighbouring β cells. We tested this possibility by inclusion of a high concentration (10 μM) of the insulin receptor antagonist S961 in the perfusion medium. As shown in Fig. 6b, S961 increased glucagon secretion by ~50%.

Very different results from those obtained in wild-type mice were obtained when insulin was tested in diabetic Fh1βKO mice. These mice lack the Krebs cycle enzyme fumarase in their β cells and develop age-dependent diabetes^32^. When severe hyperglycaemia presents (at 9–12 weeks of age), pancreatic insulin content is reduced by 97%. A clear inhibitory effect of insulin (>15% measured 2–8 min after addition of insulin) was observed in five out of seven in pancreases of hyperglycaemic Fh1βKO mice (Fig. 6c) but in none of the five experiments performed on wild-type mice (Fig. 6a; *p* < 0.03 by two-tailed Fisher’s exact test).

In intact islets, the inhibitory effects of insulin on glucagon secretion are reversed by the SSTR2 antagonist CYN154806 (Fig. 2c). CYN154806 had a similar effect when applied in the presence of insulin in perfused pancreases of diabetic Fh1βKO mice (Fig. 6d). On average, glucagon secretion increased by >100% in the presence of CYN154806. We acknowledge that this stimulation produced by the SSTR2 antagonist may not be restricted to a reversal of insulin’s glucagonostatic effect but reflects an increase in basal glucagon secretion (cf. Figure 2c).

Insulin tolerance tests confirmed that CYN154806 antagonised the hypoglycaemic effect of insulin in vivo, especially in diabetic Fh1βKO mice (compare Supplementary Fig. 9), an effect we attribute to increased glucagon secretion.

### Human islets

**Fig. 7:**
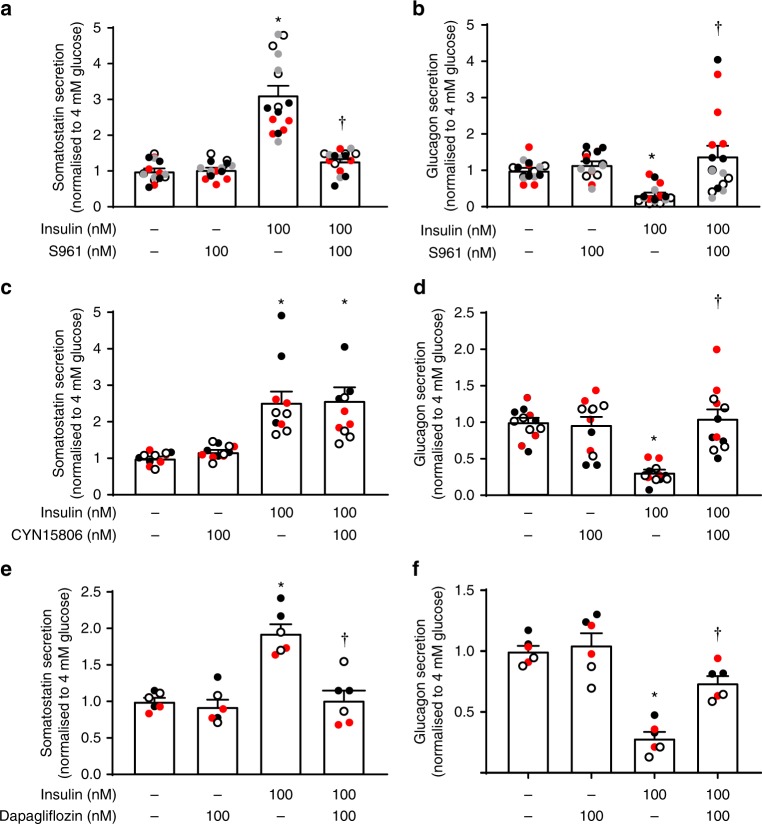
Effects of insulin in human pancreatic islets. **a**, **b** Somatostatin (**a**) and glucagon secretion (**b**) at 4 mM glucose in the absence and presence of insulin and S961 as indicated using islets from four donors (three males/one female) with four separate experiments for each donors. **c**, **d** As in **a**, **b** but testing the effects of CYN154806 as indicated (three female donors, three separate experiments for each donor). Note that CYN154806 does not affect insulin-induced somatostatin secretion but reverses the inhibitory effect of insulin on glucagon secretion. **e, f** As in **a**, **b** but instead testing the effect of dapagliflozin (100 nM; two female and one male donors, two separate experiments for each donor (different colours)). Note that dapagliflozin reduces the stimulatory and inhibitory effects of insulin on somatostatin and glucagon secretion, respectively. In **a**–**f**, data have been normalised to 4 mM glucose alone. *p < 0.01 vs 4 mM glucose; ^†^*p* < 0.01 vs 4 mM glucose and insulin, one-way ANOVA followed by Sidak’s post hoc test. In all panels, data are presented as dot plots of individual experiments and/or mean values ± S.E.M. of all experiments with the experimental series

We ascertained that the effects of insulin on somatostatin and glucagon secretion in mouse islets can be extended to human islets. In a total of 10 human islet preparations, insulin applied at 4 mM glucose stimulated somatostatin secretion by 100–200% (Fig. 7a, c, e) and inhibited glucagon secretion by >70% (Fig. 7b, d, f).

As in mouse islets, insulin’s stimulatory effect on somatostatin secretion was prevented by S961 (Fig. 7a) and dapagliflozin (Fig. 7e), effects that correlated with the abrogation of its glucagonostatic effect of insulin while not affecting glucagon secretion at 4 mM glucose alone (Fig. 7b, f). The SSTR2 antagonist had no impact on insulin’s stimulatory effect on somatostatin secretion but abolished the inhibitory effect on glucagon secretion. Unlike what was seen in mouse islets, CYN154806 did not affect glucagon secretion in islets exposed to 4 mM glucose alone (Fig. 7d, compare with Fig. 2c).

## Discussion

Here we show that insulin inhibits counter-regulatory glucagon secretion by a paracrine effect mediated by SGLT2-dependent stimulation of somatostatin release. Our data suggest that reported stimulatory effects of dapagliflozin on glucagon secretion in vivo^25,26,29,33^ and in vitro^27,28^ may reflect (at least in part) reduced somatostatin release. Importantly, cell-specific ablation of the insulin receptors in δ cells leads to the complete loss of insulin’s glucagonostatic effect and we conclude that binding of insulin to the insulin receptors on α cells does not directly inhibit glucagon secretion. From a pathophysiological viewpoint, it is of interest that exogenous insulin only affected glucagon secretion in the perfused pancreas when the experiments were conducted in diabetic animals. Thus, in non-diabetic animals/individuals, the effects of exogenous insulin on glucagon secretion are likely to be mediated by the systemic changes in plasma glucose rather than intra-islet effects of insulin. This may account for the unexpectedly mild phenotype of the α-cell-specific insulin receptor knockout mouse^34^. Islet blood flow has been reported to be from β cells to the α/δ cells^35^. Given our findings, it is of interest that exogenous insulin only inhibited glucagon secretion during retrograde infusion but was without effect anterogradely^36^.

It may seem counterintuitive that insulin retains an inhibitory effect in isolated islets (a preparation in which islet circulation has been abolished) but is without effect in the perfused pancreas (in which the islets remain vascularised). However, it should be noted that although the interstitial insulin concentration may increase to unphysiologically high levels in the centre of the isolated islet, the α and δ cells principally reside in the islet periphery in mice^17^ and they are thus exposed to an extracellular medium that is (at least initially) devoid of insulin. We acknowledge that in human islets, the α and δ cells are scattered throughout the islets^7^ but the structure is less dense than in mouse islets with the δ cells residing close to cell-free areas^37^ (possibly corresponding to blood vessels). These findings suggest that the effects of insulin on somatostatin and glucagon secretion will be critically dependent on the experimental conditions (islet isolation, incubation volume, shaking, perfusion rate, etc.), potentially explaining why insulin has previously variably been reported not to affect, stimulate or even reduce somatostatin secretion^8^.

**Fig. 8:**
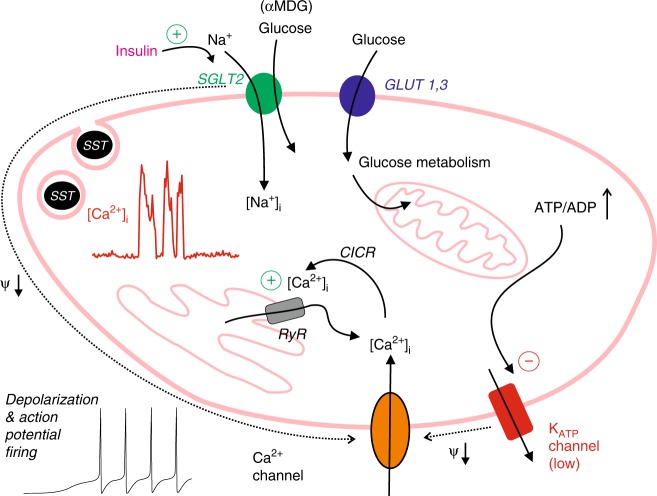
Schematic summarising the effects of insulin in δ cells. Relationship between SGLT2-mediated glucose uptake and δ-cell electrical activity, Ca^2+^ entry, Ca^2+^-induced Ca^2+^ release (CICR) and somatostatin release. Insulin stimulates SGLT2 activity. The operation of SGLT2 is electrogenic Na^+^-dependent glucose uptake mediated by SGLT2 gives rise to a small depolarising current and may (in some cells with sufficiently high SGLT2 expression and/or low K_ATP_ channel activity) trigger electrical activity (action potential firing). The associated increase in cytoplasmic Ca^2+^ ([Ca^2+^]_i_) leads to intracellular Ca^2+^ mobilisation by activation of ryanodine receptors (RyRs) and Ca^2+^-induced Ca^2+^ release (CICR). In addition to SGLT2, the δ cells also express the glucose transporters GLUT1 and GLUT3. Glucose uptake via these transporters accounts for 99% of glucose uptake. Metabolism of glucose leads to K_ATP_ channel closure, membrane depolarisation, electrical activity and CICR

Based on our experimental data, we propose the following model for insulin-induced stimulation of somatostatin secretion (Fig. 8). The δ cell possesses two glucose-sensing mechanisms that operate in parallel: one that depends on SGLT2 and one that depends on closure of the K_ATP_ channels. An increase in glucose leads to closure of the K_ATP_ channels, which produces membrane depolarisation and initiation of action potential firing in a way similar to that documented in β cells^38^. The finding that the SGLT2 inhibitor dapagliflozin is without effect on somatostatin secretion at 4 mM glucose suggests that low glucose concentrations produce little (if any) activation of the SGLT2s. This is in agreement with the finding that SGLT2 transports glucose with an affinity of 5 mM^31^. The effects of insulin on glucagon and somatostatin secretion (inhibitory and stimulatory, respectively) were abolished when extracellular Na^+^ was lowered. Using the SGLT-specific substrate αMDG, we show that insulin-induced activation of SGLT2 results in an increase in intracellular Na^+^ ([Na^+^]_i_). The operation of SGLT2 will also be associated with glucose uptake but its contribution to the overall glucose uptake is likely to be smaller compared with that mediated by GLUT1 and GLUT3, which are expressed at much higher levels. Importantly, the operation of SGLT2 is electrogenic. At low glucose (when K_ATP_ channel activity is high), the small current (~1 pA) resulting from insulin-induced operation of SGLT2 is too small to depolarise the δ cell and evoke electrical activity. Conversely, electrical activity and CICR are already maximally activated at high glucose and activation of SGLT2 will exert little additional effect. However, at 4 mM glucose, K_ATP_ channel activity is sufficiently reduced for the small insulin-induced and SGLT2-dependent current to elicit δ-cell electrical activity with resultant stimulation of Ca^2+^ influx and stimulation of CICR. This model is consistent with the observed glucose dependence of insulin’s stimulatory effect on somatostatin secretion. It also explains why insulin does not enhance somatostatin secretion evoked by tolbutamide or high-K^+^ depolarisation.

Our findings suggest that agents that reduce somatostatin action (SSTR antagonists) or secretion (SGLT2 inhibitors) might be useful as adjuncts to insulin in diabetes therapy. By restoring glucagon-mediated counter regulation, this would allow more aggressive insulin therapy (and thus improved glycaemic control) while minimising the risk of hypoglycaemia. SSTR antagonists seem particularly interesting in this respect because our in vivo data suggest that although SGLT2 inhibitors stimulate glucagon secretion, their simultaneous inhibition of glucose reabsorption dampens any hyperglycaemic actions mediated by stimulation of glucagon secretion.

## Methods

### Ethics

All experiments were conducted in compliance with all relevant local ethical regulations.

Animal experiments in the UK were conducted in accordance with the UK Animals Scientific Procedures Act (1986) and University of Oxford ethical guidelines and covered by a Home Office License. Studies in Sweden were performed after approval by the Regional Ethical Review Boards in Lund and Gothenburg.

Human pancreatic islets were obtained with ethical approval and informed consent from the Nordic Network for Islet Isolation (Uppsala, Sweden). Experiments were performed in compliance with all relevant ethical regulations and study protocols were approved by the Regional Ethical Review Board in Lund.

### Animals

Unless otherwise indicated, the hormone secretion measurements were performed using islets from female or male mice C57BL/6, 16–20 weeks of age and weighing 25–30 g.

The insulin tolerance tests in Fig. 5c, d and Supplementary Fig. 2 were performed on female or male C57Bl6 mice. The experiments in Fig. 3a, b were performed on SIRKO and control mice (generated as described below). The experiments in Supplementary Fig. 9 were performed in the non-diabetic control and diabetic Fh1βKO mice. These mice develop age-dependent progressive diabetes at 9–11 weeks of age^32^.

The electrophysiology and Ca^2+^ imaging were performed using islets from male and female mice expressing tandem-dimer red florescent protein (tdRFP, here simply referred to as RFP)  or GCaMP3 under the somatostatin promoter (see Supplementary material in ref. ^39^), respectively.

The mice were kept at constant temperature (22 °C) and 12-h light/dark cycles. They had free access to a standard chow diet (B&K) and water ad libitum.

### Generation of SIRKO mice

To generate the δ-cell-specific insulin receptor knockout mouse line (SIRKO), SST-Cre-RFP-GCaMP3 mice^40^ were crossed with homozygous insulin receptor floxed mice (IR^*fl/fl*^) (Jackson laboratory IRlox Stock Number 006955; see ref. ^41^), which have loxP sites flanking exon 4 of the insulin receptor gene. In Cre-expressing tissues, exon 4 is excised from the insulin receptor gene. If translation were to occur, a truncated protein of 308 amino acids of the N-terminal residues of the insulin receptor α-subunit, lacking the high-affinity binding site, transmembrane domain and kinase domain would putatively be produced^41^.

The first-generation offspring of the SST-Cre-RFP-GCaMP3 and IR^*fl/fl*^ mating produced SST-Cre mice heterozygous for the insulin receptor floxed allele (i.e., SST-Cre-IR^+/−^). Heterozygous SST-Cre-RFP-IR^+/−^ were selected and were back-crossed with IR^*fl/fl*^ mice to generate mice that were homozygous for deletion of the insulin receptor specifically in somatostatin-expressing tissues (including the δ cells). These are referred to as SIRKO mice (i.e., SST-Cre-RFP-IR^−/−^). It is acknowledged that there are also somatostatin-producing cells in the brain, in peripheral neurons and in the gastrointestinal tract and that insulin receptors will also be ablated in these tissues.

Littermates, which did not express SST-Cre but were homozygous or heterozygous for the floxed insulin receptor allele (IR^*fl/fl*^*or* IR^*fl/+*^), were used as controls.

To confirm knockout of the insulin receptor in δ cells, the deletion of exon 4 in the RFP-positive fraction of δ cells (obtained by  fluorescence-activated cell sorting (FACS)) was assessed by reverse transcription (RT)-PCR. To verify that no unspecific recombination occurred in other insulin receptor-expressing tissues, expression of the insulin receptor gene was assessed also in RFP-negative islets cells and in the liver (as negative controls). To generate complementary DNA (cDNA), ~10 ng of total RNA (extracted from FAC-sorted RFP-positive islet cells, RFP-negative islet cells and from the liver of each SIRKO mouse) was reverse transcribed.

PCR from corresponding cDNA yielded an amplicon of 514 bp in the control tissues and an amplicon of 365 bp (lacking exon 4) in the SST-Cre-expressing (RFP-positive) cells of SIRKO mice. To verify that the 365-bp band corresponded to the truncated transcript, whereas the 514-bp corresponded to the wild-type copy, the fragments were cloned into a pGEM®-T Easy vector (Promega) and sequenced.

### Preparation of pancreatic islets

Mice were killed by cervical dislocation, the pancreases quickly removed and islets isolated either by collagenase (Sigma) or liberase (Roche) digestion.

### Media

For historical and technical reasons, slightly different media were used for the various experiments.

For the hormone secretion measurements, a Krebs Ringer bicarbonate buffer composed of (mM) 120 NaCl, 3.6 (human) or 4.7 (mouse) KCl, 2.5 CaCl_2_, 1.2 KH_2_PO_4_, 1.2 MgSO_4_, 10 HEPES and 25 NaHCO_3_ (pH 7.4 with NaOH) supplemented with 0.2% (weight/volume) bovine serum albumin (BSA) was used (EC1).

During the Ca^2+^ imaging experiments, the islets were superfused with a buffer consisting of (mM) 139 NaCl, 4.7 KCl, 0.5 MgSO_4_, 2.6 CaCl_2_, 5 HEPES (pH 7.40), 0.5 NaH_2_PO_4_ and 2 NaHCO_3_ (EC2).

In the membrane potential recordings, the extracellular contained (mM) 140 NaCl, 3.6 KCl, 0.5 MgSO_4_, 0.5 NaH_2_PO_4_, 2 NaHCO_3_, 5 HEPES, 1.5 CaCl_2_ (EC3). The pipette solution contained (mM) 76 K_2_SO_4_, 10 NaCl, 10 KCl, 1 MgCl_2_, 5 HEPES (pH 7.35 with KOH) (IC1) and 60 μg/ml of the pore-forming antibiotic amphotericin B.

The measurements of cytoplasmic Na^+^ were performed in a medium containing (mM) 140 NaCl, 4.7 KCl, 2.6 CaCl_2_, 1.2 MgCl_2_, 1 NaH_2_PO_4_, 5 NaHCO_3_, 10 HEPES and glucose as indicated (pH 7.4, with NaOH) (EC4).

All media were supplemented with glucose as indicated.

Whenever Na^+^ was reduced, iso-osmolarity was maintained by adding an equal amount of choline-Cl (Na^+^). The measured osmolality of the media ranged between 310 and 315 mOsm.

IGF-1, the SSTR2 antagonist CYN154806 and the IGF-1 receptor antagonist PQ401 were obtained from Tocris. All other chemicals were from Merck AG, (Darmstadt, Germany) or Sigma (USA).

### Hormone release measurements in mouse islets

In each secretion experiment, groups of islets were pooled together and 12 size-matched islets from 4 to 6 mice were preincubated for 30 min at 37 °C in 1 ml of EC1 gassed with 95/5% O_2_/CO_2_ and supplemented with 0.1% fatty acid-free bovine serum albumin, and 1 mM glucose.

After preincubation, the buffer was changed to a medium supplemented with test agents, and the islets were incubated for 60 min. All incubations were performed at 37 °C with gentle shaking (30 cycles/min).

Immediately after incubation, aliquots of the medium were removed for assay of glucagon and somatostatin.

The radioimmunoassay kits for glucagon and somatostatin were from Millipore (USA) and Eurodiagnostica (Malmö, Sweden), respectively.

The accuracy of the size matching was confirmed by measurements of islet hormone content in a few of the experiments. The coefficient of variation was 24 ± 8% (mean ± S.D. for four experiments) for somatostatin and 27 ± 8% (*n* = 9) for glucagon.

### Perfused mouse pancreas

The mouse pancreas perfusion experiments were performed as described previously^42^. Briefly, the pancreas was perfused at the physiological rate in the presence of 4 mM glucose.

### Hormone release measurements in human islets

For experiments on human islets, the islets had been cultured in CMRL 1066 (ICN Biomedicals, Costa Mesa, CA) supplemented with 10 mM HEPES, 2 mM l-glutamine, 50 μg/ml gentamicin, 0.25 μg/ml fungizone (Gibco, BRL, Gaithersburg, MD), 20 μg/ml ciprofloxacin (Bayer Healthcare, Leverkusen, Germany) and 10 mM nicotinamide at 37 °C (5% CO_2_) for 1–5 days prior to the experiments. The glucose concentration of the CMRL 1066 medium is 5 mM.

The incubations were carried out essentially as described for mouse islets above and as reported previously^37,43,44^ using the same reagents and assays.

The accuracy of the size matching was confirmed by measurements of islet hormone content. The coefficient of variation was 28 ± 12% (mean ± S.D. for 16 experiments) for somatostatin and 28 ± 8% (*n* = 16) for glucagon.

### [Ca^2+^]_i_ imaging

Confocal [Ca^2+^]_i_ imaging in islets from SST-GCaMP3 mice was conducted in EC2 essentially as previously reported^42^. GCaMP3 was excited at 488 nm, and fluorescence emission recorded at 510–515 nm. The pinhole diameter was kept constant, and frames of 256 × 256 pixels were taken every 1–3 s. Images were acquired using ZenBlue software (Carl Zeiss).

### Membrane potential recordings

Membrane potential recordings were performed using EC3 and IC1 at 34 °C using the perforated-patch technique as described previously^45^. Mouse δ cells were identified by the expression of tdRFP or GCaMP3.

### Intracellular Na^+^ measurements

Time-lapse imaging of [Na^+^]_i_ in dispersed mouse islets was performed on a Zeiss Axiozoom.V16 microscope. Cells were pre-loaded with 6 μM of Sodium Green (Molecular Probes) for 30 min at room temperature and imaged at several locations simultaneously. Sodium Green was excited at 490 nm and emission was collected at 515 nm, using a CCD camera. Time-lapse images were collected every 60 s and the bath solution (EC4) was superfused at 60 μl/min and the temperature maintained at 34 °C. δ Cells were identified by the RFP fluorescence. Images were acquired using ZenBlue software (Carl Zeiss).

### Flow cytometry of islet cells (FACS)

Pancreatic islets from either SST-Cre-GCaMP3, or SST-Cre-RFP, or SIRKO mice as described above were dissociated into single cells by trypsin digestion and mechanical dissociation. Briefly, following isolation islets were incubated in 1 ml of trypsin (TrypLE, Gibco) at 37 °C for 5 min. Enzymatic digestion was stopped by adding 9 ml of RPMI-1640 medium supplemented with 10% fetal serum albumin (FBS), 1% penicillin–streptomycin and 5 mM glucose. The islets/islet fragments were centrifuged for 5 min at 400 g at room temperature. The supernatant was removed, and the islet pellet was resuspended in 600 μl of RPMI-1640 supplemented with 2% FBS, 1% penicillin–streptomycin and 10 mM glucose. The trypsin-treated islets were mechanically dissociated into single cells by trituration and filtered through a 30-μm filter to remove remaining clumps of cells.

Single cells were passed through a MoFlo Legacy (Beckman Coulter). Fractions of GCaMP3- or RFP-positive cells were isolated by combining several narrow gates. Forward and side scatter were used to isolate small cells and to exclude cell debris. Cells were then gated on pulse width to exclude doublets or triplets. GCaMP3-positive cells were excited with a 488 nm laser and the fluorescent signal was detected through a 530/40 nm bandpass filter (i.e., in the range 510–550 nm). RFP was excited with the 488 nm laser and the fluorescent signal was detected through a 580/30 nm bandpass filter (i.e., in the range 565–595 nm). GCaMP3- or RFP-negative cells were collected in parallel (Supplementary Fig. 1).

### Plasma glucose measurements and insulin tolerance tests

Fed blood glucose levels (data point before fasting) were measured from a blood drop obtained by a tail vein nick using the Accu-Chek Aviva (Roche Diagnostic). The mice were then fasted for 3–4 h prior to the experiments. Fast-acting human insulin (Actrapid, Novo Nordisk) was injected intraperitoneally at the indicated doses with a 25-gauge needle at time zero. CYN154806 or dapagliflozin were injected intraperitoneally with insulin as indicated. In the control experiments, insulin was co-injected with the solvent.

Tail vein blood glucose levels were monitored using a glucometer before and 15, 30, 45, 60 and 90 min after injection and samples for serum glucagon analysis (Mercodia Glucagon ELISA, Uppsala, Sweden) were collected in some experiments.

The hypoglycaemic effect of insulin varies depending on the mouse strain, age of the animals and the experimenter, which is why the hypoglycaemic effect of insulin shows some variability. Any statistical analyses are based on animals of the same age and strain and experiments conducted by the same person.

### Immunocytochemistry

For immunocytochemistry, single cells were fixed in 2.5% paraformaldehyde and kept at 4 °C. On the day of the experiment, cells were permeabilised with 0.1% Triton X-100 in phosphate-buffered saline (PBS) for 5 min at room temperature. Permeabilisation was followed by blocking of nonspecific binding with PBS containing 5% swine serum for 20 min. The cells were then incubated overnight with anti-SGLT2 antibodies (1:75, Santa Cruz Sc-393350) at 4 °C. On day 2, cells were incubated with mouse Alexa-488 Tyramide kit (T20948, Molecular Probes) according to manufacturer’s instruction. Cells were then incubated in primary antibodies anti-somatostatin (1:100, Millipore, MAB354) for 1 h at room temperature, followed by secondary antibodies Alexa 568 anti-rat (1:500, A-11077, Invitrogen) for 30 min by the nuclear stain RedDot2 (1:200, 40061, Biotium) for 10 min at room temperature. Samples were visualised with a Bio-Rad confocal microscope using appropriate lasers and filters.

### Data analysis

The action potential frequency was analysed after exporting the Pulse files as ASCII files and conversion into an axon binary file (using ABF File Utility, v2.1.57, Synaptosoft Inc., Fort Lee, NJ). The recordings were then analysed in Clampfit 9 (Molecular Devices, Sunnyvale, CA). A template was generated for each individual experiment by averaging 10–20 action potentials from each recording. Action potentials were then detected and analysed using the event detection/template search function of Clampfit 9.

For the analysis of the Ca^2+^ imaging, the recordings were replayed off-line, and regions of interest were selected and analysed using the Zeiss LSM510 and Zen Blck software and Origin 8.5 (OriginLab Corporation, Northampton, MA, USA). Fluorescence signals (F) have been normalised to initial fluorescence (F_0_). The frequencies were calculated as numbers of Ca^2+^ spikes divided by the duration of each condition.

Unless otherwise indicated, data are given as mean values ± standard error of the mean (S.E.M.) of the indicated number of experiments (*n*). In the histograms, the data points for the individual experiments are shown. For clarity, only mean values ± S.E.M. are presented in the graphs.

In the hormone secretion measurements, each group of 12 islets was counted as an experiment (islets pooled from 3 to 8 different mice). Experiments were normally performed on 2–3 different days with new media prepared for each day.

For hormone secretion measurements on human islets, each group of 12 islets was counted as an experiment. Experiments were performed using islets from three donors. For the studies described in Fig. 7, islets from a total of 10 different donors were used.

Because the experiments have been performed over >10 years and by several investigators, secretion has been normalised to basal (usually 4 mM glucose, unless otherwise indicated).

In the electrophysiology, Ca^2+^ and Na^+^ imaging, each cell was counted as an experiment but cells were taken from multiple islets and animals.

Statistical significances were (unless otherwise stated) evaluated using Student’s *t*-test or analysis of variance (ANOVA) followed by appropriate post hoc tests for multiple comparisons.

### Reporting summary

Further information on experimental design is available in the Nature Research Reporting Summary linked to this article.

## Supplementary information

Supplementary Information

Reporting Summary

## Data Availability

The authors declare that all data supporting the findings of this study are available within the article and its Supplementary Information Files or from the corresponding author on reasonable request. A reporting summary for this Article file is available as a Supplementary Information file.
