# Supplementary material for: Insulin inhibits glucagon release by SGLT2-induced stimulation of somatostatin secretion
Source: Nat Commun. 2019 Jan 11;10:139. doi: 10.1038/s41467-018-08193-8 (PMC6329806; doi:10.1038/s41467-018-08193-8)
Supplement: Supplementary file 1 — Supplementary Information [file 41467_2018_8193_MOESM1_ESM.docx]

**Supplementary Information**

Insulin inhibits glucagon release by SGLT2-induced stimulation of somatostatin secretion

**Elisa Vergari, Jakob G. Knudsen, Reshma Ramracheya, Albert Salehi,**

**Quan Zhang, Julie Adam, Ingrid Wernstedt Asterholm^,^ Anna Benrick,**

**Linford J. B. Briant, Margarita V. Chibalina, Fiona M. Gribble,**

**Alexander Hamilton, Benoit Hastoy, Frank Reimann, Nils J. G. Rorsman, Ioannis I. Spiliotis, Andrei Tarasov,** **Yanling Wu,** **Frances M. Ashcroft**

**and Patrik Rorsman**

**
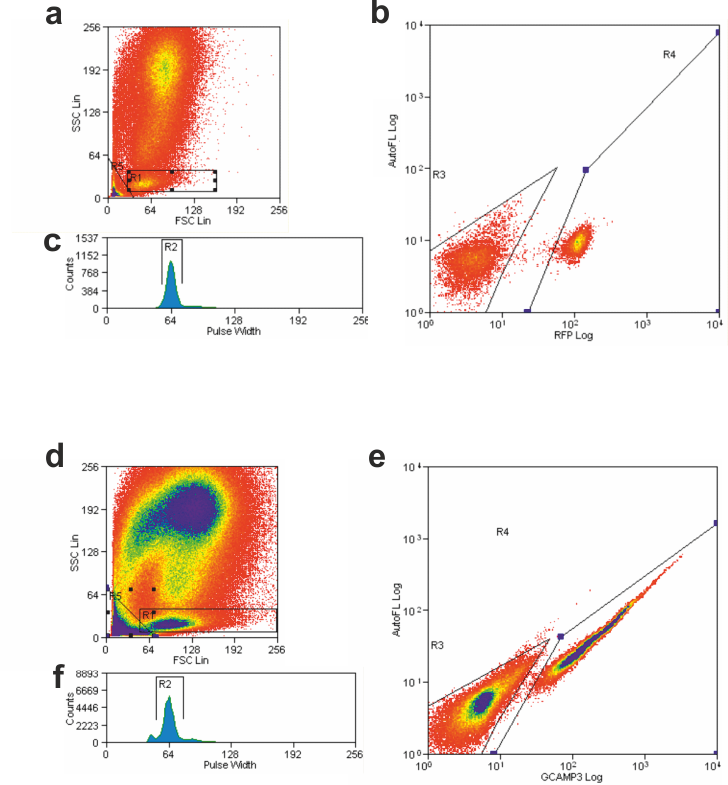
**

**Supplementary Fig. 1.** FACS analysis of SST-Cre-RFP and SST-Cre-GCaMP3 cells. **(a-c)** Small cells were selected using R1 gate for forward and side scatter (a). RFP-positive cells (gated in R4) were collected separately from RFP negative cells (gated in R3) (b). To exclude doublets or triplets cells were then gated on pulse width R2 (c). (**d-f**) As in (a-c) but GCaMP3 positive cells were sorted.

**
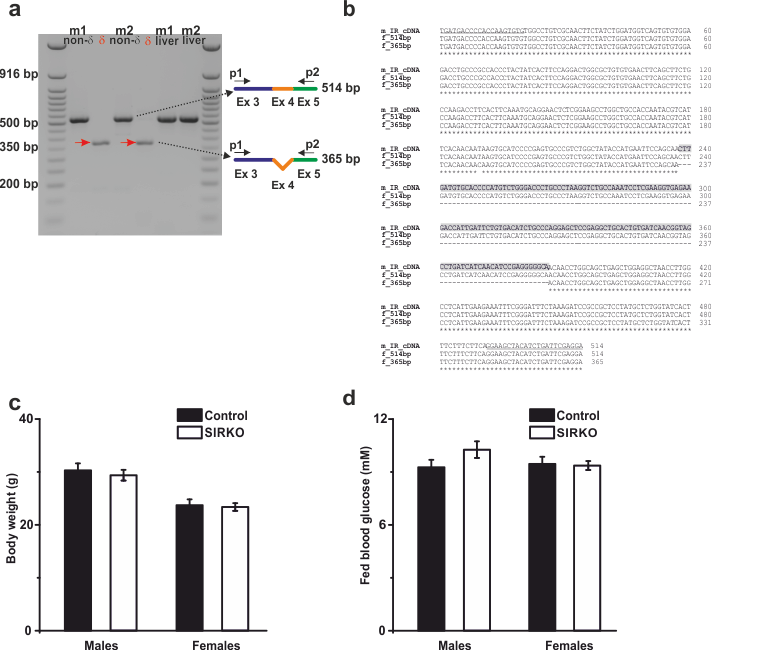
Supplementary Fig. 2.** Generation of δ-cell-specific insulin receptor knockout mice (SIRKO). (**a**) Agarose gel showing a ~365bp low molecular weight PCR product obtained from cDNA of δ-cells (δ) isolated by FACS from islets from two SIRKO mice (m1, m2). The PCR products from non-δ-cells (islet α- and β-cells) and the liver yielded a higher molecular weight band ~514bp (lacking in SIRKO δ-cells), corresponding to the wild-type insulin receptor gene. (**b**) Nucleotide alignment performed using Kalign (http://www.ebi.ac.uk/Tools/msa/kalign/). Top line (m_IR_cDNA) is the cDNA sequence for the mouse insulin receptor (NM_010568.3) from nucleotide 1227 to 1740 (numbered 1-514 in the figure). Middle line (f_514bp) corresponds to the sequence of the cDNA obtained from SIRKO non-δ-cells. Bottom line (f_365bp) is the cDNA sequence obtained from SIRKO δ-cells. Sequences of the primers used for PCR are underlined. The highlighted sequence corresponds to the sequence of the exon 4 (149bp), which is absent in the cDNA obtained from δ-cells. (**c**) Body weight of male and female control and SIRKO mice (n= 12 per group). (**d**) As in (c) but fed plasma glucose levels (n=12). Data in (c-d) are mean values ± S.E.M.

**
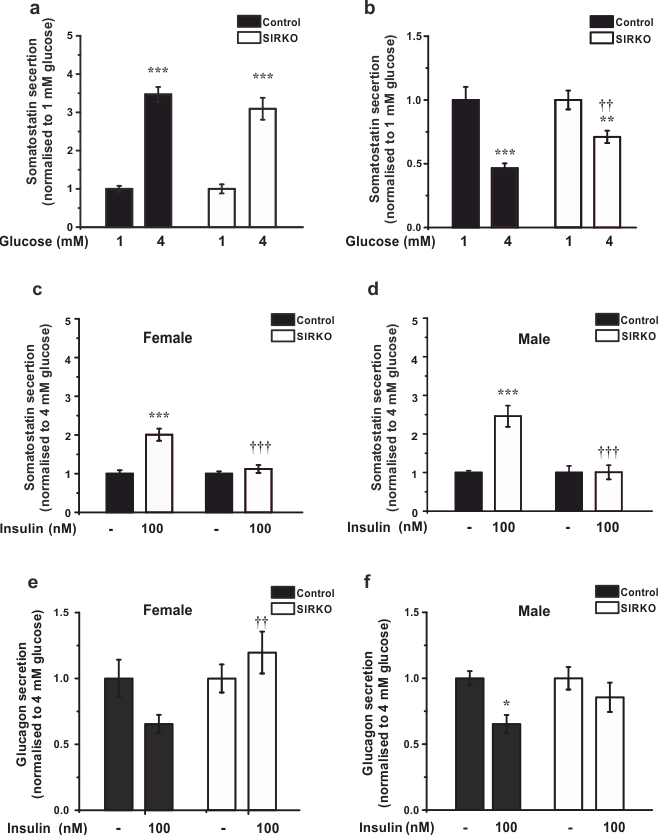
**

**Supplementary Fig. 3.** Characterization of SIRKO mice. (**a-b**) Effects of increasing glucose from 1 to 4 mM on somatostatin (**a**) and glucagon (**b**) secretion in control (CTRL) and SIRKO **i**slets. ***p*<0.01; ****p*<0.001 vs 1 mM glucose in the same mouse strain; ^††^*p*<0.01 vs 4 mM glucose in CTRL islets (n=12-18 experiments/4 mice). (**c-d**) Somatostatin secretion in the absence and presence of insulin as indicated in female (**c**) and male (**d**) control and SIRKO mice. ^††^*P*<0.01 vs 4 mM glucose in CTRL islets (n=6-10 experiments/4 mice for each sex/strain), one-way ANOVA followed by Dunnett’s posthoc test. (**e-f**) As in (c-d) but glucagon secretion was measured. ^††^*p*<0.01 vs 4 mM glucose in CTRL islets (n=6-10 experiments/4 mice for each sex/strain), one-way ANOVA followed by Dunnett’s posthoc test. Responses have been normalized to secretion at 1 mM glucose (**a-b**) and 4 mM glucose (**c-f**) as indicated. Data in are mean values ± S.E.M.


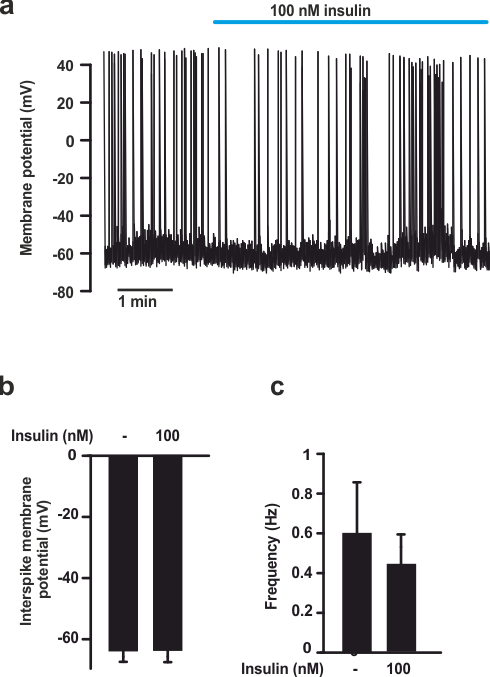


**Supplementary Fig. 4.** No effects of insulin on δ-cell electrical activity. (**a**) Membrane potential recording from a δ-cell in an intact pancreatic islet at 4 mM glucose before, during and after application of insulin (100 nM; indicated by horizontal line). (**b-c**) Histograms summarizing effect of insulin on interspike membrane potential (b) and action potential frequency (c) at 4 mM glucose (n=7 cells from 3 mice). Data in (b-c) are mean values ± S.E.M.

**
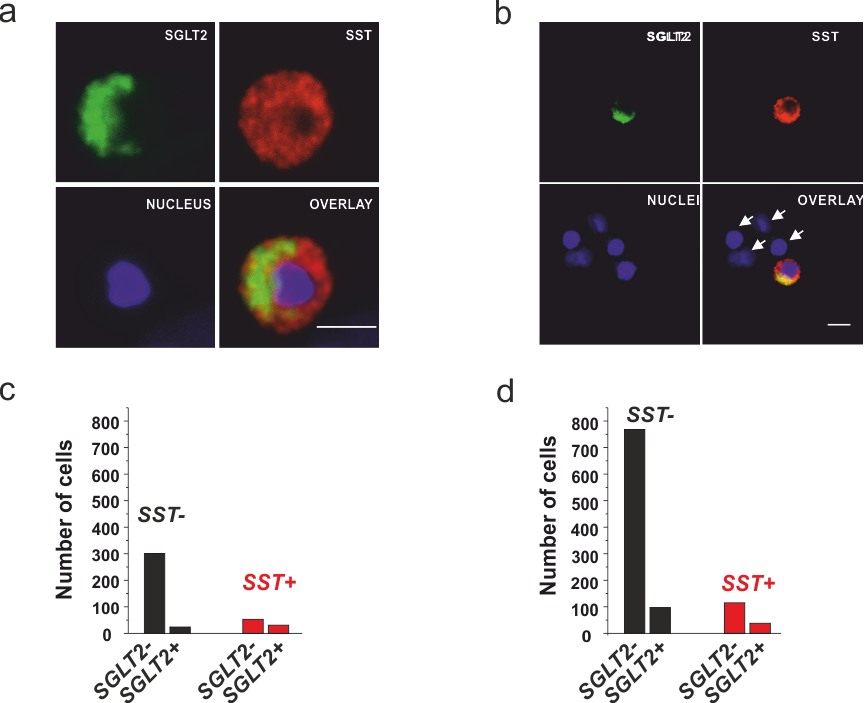
**

**Supplementary Fig. 5.** SGLT2 expression in mouse and human δ-cells. **(a)** Expression of SGLT2 in dispersed mouse islet cells. Immunocytochemical detection of SGLT2 (green), somatostatin (SST; red) and the overlay of the two (yellow). The nuclei have been labelled using the far-red stain Red dot (blue). (**b**) As in (**a**) but in human islet cells. The arrows highlight four cells (identified by nuclear stain) that were somatostatin- and SGLT2-negative. Scale bars: 10 μm. (**c-d**) Number of SGLT2-positive non-δ-cells and δ-cells (somatostatin-positive) in cultures of dissociated mouse (**c**) and human islet cells (**d**). Data from 3 different mice (females) or donors of both sexes.

**
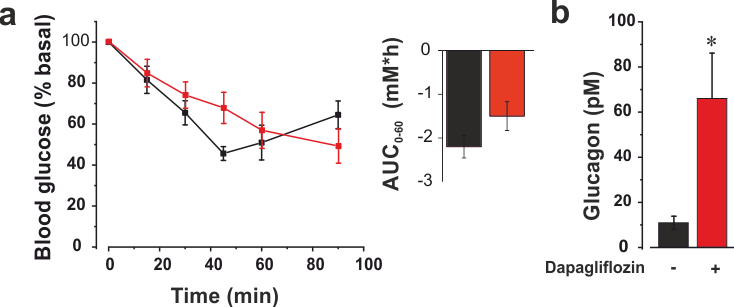
**

**Supplementary Fig. 6.** Effects of dapagliflozin on plasma glucose and glucagon during insulin-induced hypoglycaemia. (**a**) Plasma concentrations following intraperitoneal (ip) injection of insulin (at t=0; 0.5 U/kg) in female mice (age: 16 wks) followed by injection of dapagliflozin (10 mg/kg, ip: red) or vehicle (5% DMSO in sterile PBS, ip; black). Inset summarises effects of dapagliflozin or plasma glucose expressed as area under the curve (AUC) during the first 45 min after injection of insulin. (**b**) Plasma glucagon measured in the presence of insulin at t=90 min in the absence and presence of dapagliflozin as indicated. There was no difference in plasma glucagon at t=0. **p*<0.05 vs control. Student’s *t*-test. (4 mice for each condition). Data in are mean values ± S.E.M.

**
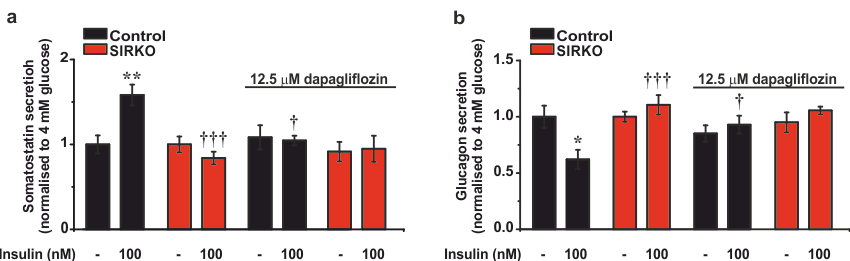
**

**Supplementary Fig. 7.** Effects of insulin and dapagliflozin on somatostatin and glucagon secretion require the presence of δ-cell insulin receptors. (**a-b**) Somatostatin (**a**) and glucagon secretion (**b**) at 4 mM glucose in the absence or presence of insulin and in absence or presence of dapagliflozin in islets from control and SIRKO mice. **p*<0.05 and ***p*<0.01 vs 4 mM glucose in control islets; ^†^*p*<0.05 and ^†††^ *p*<0.001 vs 4 mM glucose in the presence of insulin in control islets (n=5-6 experiments/4 mice of both sexes for each strain), one-way ANOVA followed by Dunnett’s posthoc test. Data in (c-d) are mean values ± S.E.M.

**
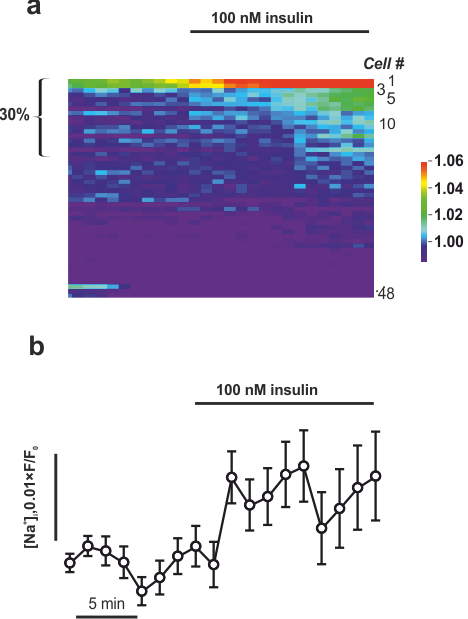
**

**Supplementary Fig. 8.** Effects of insulin on cytoplasmic Na^+^ ([Na^+^]_i_). (**a**) Sodium Green fluorescence (displayed in pseudocolours) measured in 48 different δ-cells simultaneously (each cell represented by a horizontal line). Each line represents an individual cell. Note that insulin effect in ~30% of the cells. (**b**) Average response in all 48 cells. Data represent Sodium Green fluorescence and have been normalised to initial value (F_0_). Insulin produces a statistically significant increase in [Na^+^]_i_ (*p*<0.05 by paired repeated means ANOVA). Data in (b) are mean values ± S.E.M.

**
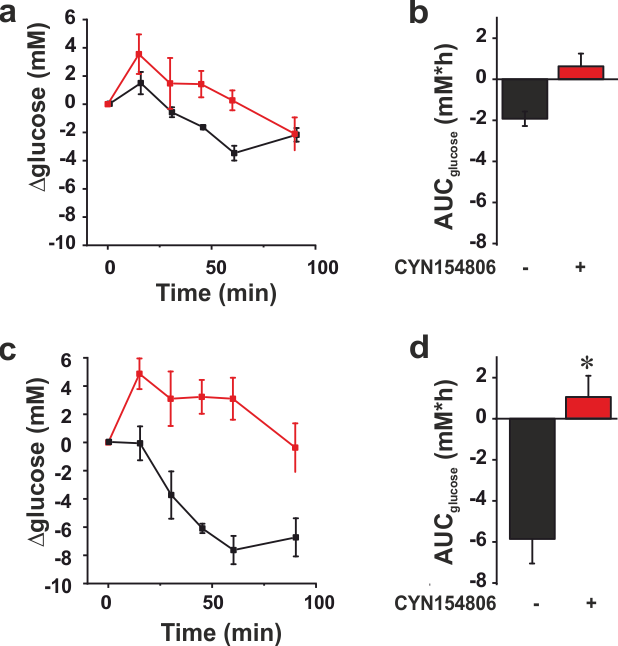
**

**Supplementary Fig. 9.** (**a,c**) Insulin tolerance test in control (a) and diabetic Fh1βKO mice of both sexes (c) in the absence (black) and presence of CYN154806 (red). Plasma glucose concentrations following injection of insulin (at t=0; 0.75 U/kg body weight) under control conditions (black) and in the presence (red) of the SSTR2 antagonist CYN1548006 (0.5 mg/kg body weight). The net changes in plasma glucose (Δglucose) are plotted. Plasma glucose at t=0 (after 4 hours of fasting) averaged 9±1 (n=6) and 19±1 mM (n=7) in control and Fh1bKO mice, respectively. (**b,d**) Area under the curve for the insulin-induced reduction of plasma glucose during the first 60 min in the absence (black) and presence of CYN154806 (red) as indicated in control (b) and hyperglycaemic Fh1bKO mice (d), respectively. **p*<0.01 vs no CYN154806 (Student’s *t*-test). Data are mean values ± S.E.M.
