# Supplementary material for: Insulin inhibits glucagon release by SGLT2-induced stimulation of somatostatin secretion
Source: Nat Commun. 2019 Jan 11;10:139. doi: 10.1038/s41467-018-08193-8 (PMC6329806; doi:10.1038/s41467-018-08193-8)
Supplement: Supplementary file 2 — Reporting Summary [file 41467_2018_8193_MOESM2_ESM.pdf]

nature research

Corresponding author(s): Patrik Rorsman

Reporting Summary

Nature Research wishes to improve the reproducibility of the work that we publish. This form provides structure for consistency and transparency in reporting. For further information on Nature Research policies, see Authors & Referees and the Editorial Policy Checklist.

Statistical parameters

When statistical analyses are reported, confirm that the following items are present in the relevant location (e.g. figure legend, table legend, main text, or Methods section).

n/a	Confirmed	
☐	☒	The exact sample size (n) for each experimental group/condition, given as a discrete number and unit of measurement
☐	☒	An indication of whether measurements were taken from distinct samples or whether the same sample was measured repeatedly
☐	☒	The statistical test(s) used AND whether they are one- or two-sided<br>*Only common tests should be described solely by name; describe more complex techniques in the Methods section.*
☐	☒	A description of all covariates tested
☐	☒	A description of any assumptions or corrections, such as tests of normality and adjustment for multiple comparisons
☐	☒	A full description of the statistics including central tendency (e.g. means) or other basic estimates (e.g. regression coefficient) AND variation (e.g. standard deviation) or associated estimates of uncertainty (e.g. confidence intervals)
☐	☒	For null hypothesis testing, the test statistic (e.g. F, t, r) with confidence intervals, effect sizes, degrees of freedom and P value noted<br>*Give P values as exact values whenever suitable.*
☒	☐	For Bayesian analysis, information on the choice of priors and Markov chain Monte Carlo settings
☒	☐	For hierarchical and complex designs, identification of the appropriate level for tests and full reporting of outcomes
☒	☐	Estimates of effect sizes (e.g. Cohen's d, Pearson's r), indicating how they were calculated
☐	☒	Clearly defined error bars<br>*State explicitly what error bars represent (e.g. SD, SE, CI)*

Our web collection on statistics for biologists may be useful.

Software and code

Policy information about availability of computer code

Data collection	ZenBlue (Carl Zeiss)<br>ZenBlack (Carl Zeiss)<br>Pulse or Patchmaster(HEKA)
Data analysis	Origin 8.5<br>Synaptosoft<br>GraphPad Prism 6.0<br>ImageJ<br>Clampfit<br>Excel

For manuscripts utilizing custom algorithms or software that are central to the research but not yet described in published literature, software must be made available to editors/reviewers upon request. We strongly encourage code deposition in a community repository (e.g. GitHub). See the Nature Research guidelines for submitting code & software for further information.

Data

Policy information about availability of data

All manuscripts must include a data availability statement. This statement should provide the following information, where applicable:

- Accession codes, unique identifiers, or web links for publicly available datasets
- A list of figures that have associated raw data
- A description of any restrictions on data availability

The authors declare that all data supporting the findings of this study are available within the article and its Supplementary Information Files or from the corresponding author on reasonable request.

Field-specific reporting

Please select the best fit for your research. If you are not sure, read the appropriate sections before making your selection.

☒ Life sciences ☐ Behavioural & social sciences ☐ Ecological, evolutionary & environmental sciences

For a reference copy of the document with all sections, see nature.com/authors/policies/ReportingSummary-flat.pdf

Life sciences study design

All studies must disclose on these points even when the disclosure is negative.

Sample size	8 or more experiments were performed using pancreatic islets from at least 3 different mice of (for studies of human tissues) 3 or more donors
Data exclusions	No data were excluded but some experiments were excluded based on technical grounds when it was evident from internal control conditions that cells/tissues were not functioning.
Replication	Effects of insulin have been replicated on multiple occasions over >10 years and by at least two different investigators working in different laboratories and different countries
Randomization	Not relevant because each experiment involved its own control and test contions
Blinding	Blinding was not possible in most experiments because experiments involved testing of several conditions. For studies in transgenic animals, the person conducting the experiment was not aware of the mouse genotype during the experiment.

Reporting for specific materials, systems and methods

Materials & experimental systems

n/a	Involved in the study	
☐	☒	Unique biological materials
☐	☒	Antibodies
☒	☐	Eukaryotic cell lines
☒	☐	Palaeontology
☐	☒	Animals and other organisms
☒	☐	Human research participants

Methods

n/a	Involved in the study	
☒	☐	ChIP-seq
☐	☒	Flow cytometry
☒	☐	MRI-based neuroimaging

Unique biological materials

Policy information about availability of materials

Obtaining unique materials	The SIRKO mouse was generated as described and has been cryopreserved. It will be made available on request.<br><br>All other materials are obtaineble from commercial suppliers

Antibodies

Antibodies used

anti-SGLT2 (Santa Cruz, Sc-393350)
anti-somatostatin (Millipore, MAB354)

Alexa-488 (Molecular Probes T20948)
Alexa 568 anti rat (Invitrogen A-11077)

Validation

The anti-SGLT2 was used as previously validated by Bonner et al. nature Medicine 2015.

The anti-somatostain antibody as reported on millipore website recognizes somatostatin and shows no cross-reactivity to enkephalins, other endorphins, substance P or CGRP. It partially cross-reacts with somatostatin fragments. Millipore website stated that it worked on immunohistochemistry at 1:50-1:100 on human brain. Our optimal working dilution was determined to be 1:75.

Animals and other organisms

Policy information about studies involving animals; ARRIVE guidelines recommended for reporting animal research

Laboratory animals

C57Bl6 (females and males as indicated), delta-cell insulin receptor knockoyt mice (SIRKO) and mice expressing the genetically encoded calcium sensor GCamP3 in somatostatin-producing cells

Wild animals

N/A

Field-collected samples

N/A

Flow Cytometry

Plots

Confirm that:

☒ The axis labels state the marker and fluorochrome used (e.g. CD4-FITC).

☒ The axis scales are clearly visible. Include numbers along axes only for bottom left plot of group (a 'group' is an analysis of identical markers).

☒ All plots are contour plots with outliers or pseudocolor plots.

☒ A numerical value for number of cells or percentage (with statistics) is provided.

Methodology

Sample preparation

Pancreatic islets from either SST-Cre-GCaMP3, or SST-Cre-RFP, or SIRKO mice were dissociated into single cells by trypsin digestion and mechanical dissociation. Briefly, following isolation as described above, the islets were incubated in 1 ml of trypsin (TrypLE, Gibco) at 37 ºC for 5 min. Enzymatic digestion was stopped by adding 9 ml of RPMI 1640 medium supplemented with 10% FBS, 1% penicillin-streptomycin and 5 mM glucose. The islets were centrifuged for 5 min at 400 g at room temperature. The supernatant was removed, and the islet pellet was re-suspended in 600 µl of RPMI 1640 supplemented with 2% FBS, 1% penicillin-streptomycin and 10 mM glucose. The islets were mechanically dissociated into single cells by trituration and filtered through a 30 µm filter to remove remaining clumps of cells. Single cells were passed through a FAC sorter (MoFlo Legacy from Beckman Coulter)

Instrument

Beckman Coulter Legacy MoFlo MLS High Speed Cell Sorter

Software

PC-based (WinXP Pro) digital compensation software (Summit 4.3)

Cell population abundance

The abundance of RFP-positive cells or GCaMP3-positive cells varied between 1.5-2.5% of the total cell-sorted population. Purity was determined post-sorting by quantifying insulin, glucagon and somatostatin expression in sorted and non-sorted population by qPCR.

Gating strategy

GCaMP3 or RFP-positive cells were purified by combining several narrow gates. Forward and side scatter were used to isolate small cells (R1) and to exclude cell debris (R5). Cells were then gated on pulse width to exclude doublets or triplets (R2). GCaMP3 positive cells were excited with a 488 nm laser and the fluorescent signal was detected through a 530/40 bandpass filter (i.e. in the range 510-550 nm). RFP was excited with the 488 nm laser and the fluorescent signal was detected through a 580/30 bandpass filter (i.e. in the range 565-595 nm) (R4). GCaMP3- or RFP-negative cells were collected separately (R3).

☒ Tick this box to confirm that a figure exemplifying the gating strategy is provided in the Supplementary Information.
